# Supplementary material for: Integrative molecular characterization of sarcomatoid and rhabdoid renal cell carcinoma
Source: Nat Commun. 2021 Feb 5;12:808. doi: 10.1038/s41467-021-21068-9 (PMC7865061; doi:10.1038/s41467-021-21068-9)
Supplement: Supplementary file 1 — Supplementary Information [file 41467_2021_21068_MOESM1_ESM.pdf]

Integrative Molecular Characterization of Sarcomatoid and Rhabdoid Renal Cell Carcinoma

Bakouny Z. et al.

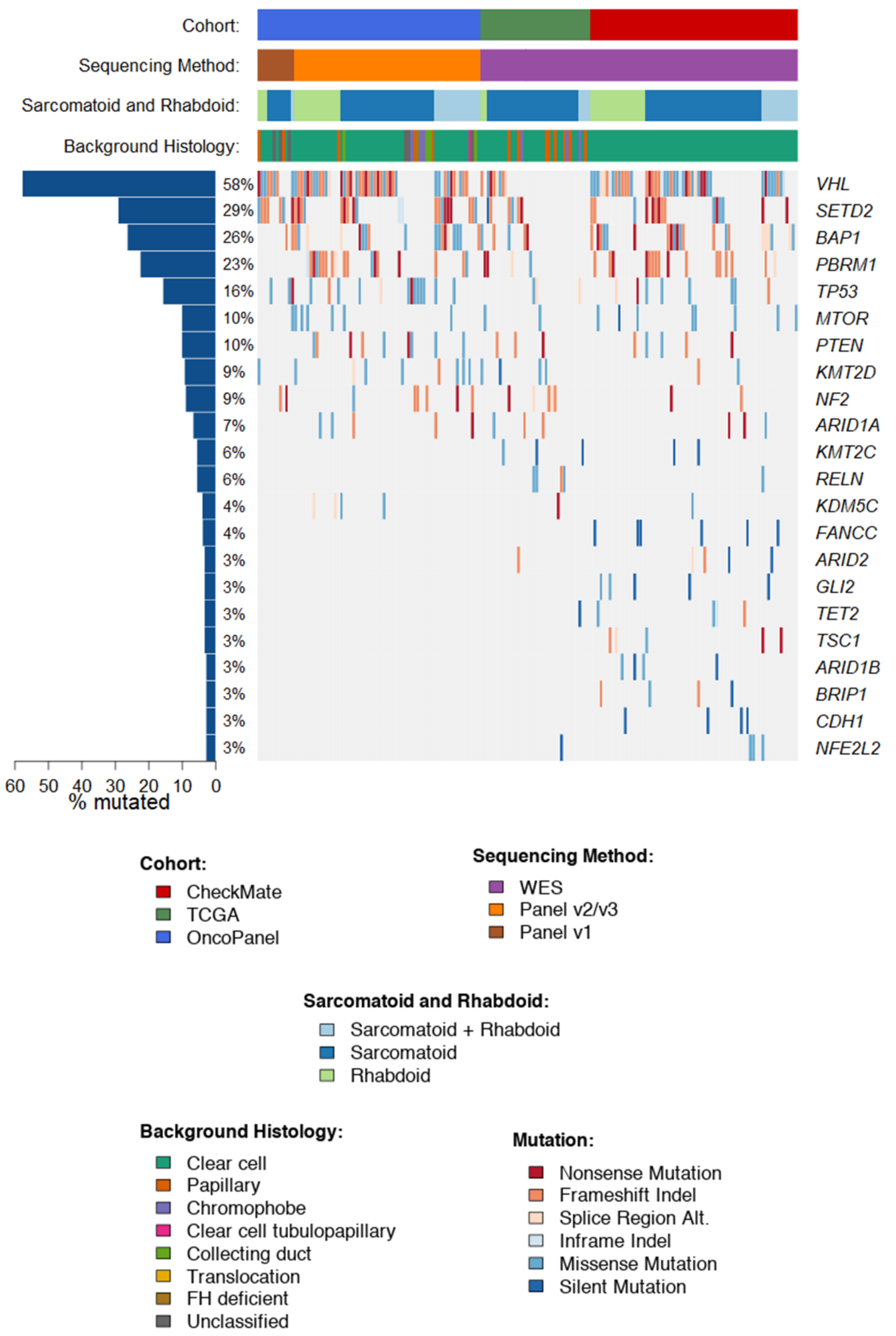

Figure S1: Co-mutation plot of patients with S/R RCC across the CheckMate, OncoPanel, and TCGA cohorts (in relation to Fig. 1). OncoPanel (all versions) did not include *KMT2C* or *RELN*. OncoPanel v1 did not include *KDM5C*, *KMT2D*, or *PBRM1* genes. The percentage mutated numbers take this into account by excluding the corresponding patients from the percentage calculation. Alt: Alteration; TCGA: The Cancer Genome Atlas; WES: Whole Exome Sequencing.

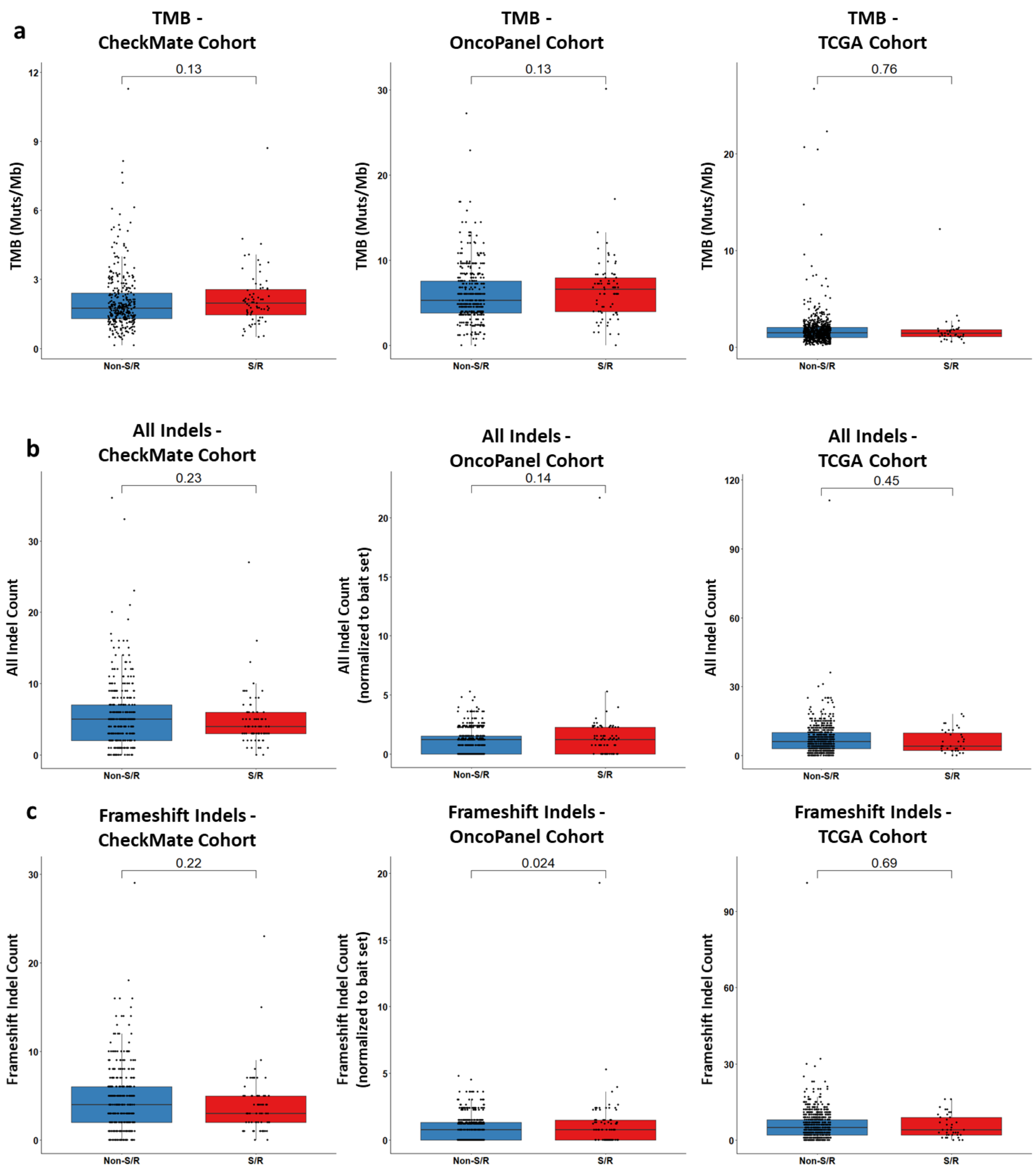

Figure S2: Mutation and indel burden of S/R RCC tumors (in relation to Fig. 1). S/R RCC tumors have a similar overall (a) tumor mutational burden, (b) total indel load, and (c) frameshift indel load compared to non-S/R RCC tumors in the CheckMate, TCGA, and OncoPanel cohorts. Two-sided Mann-Whitney U test p-values shown. CheckMate (N= 69 S/R and N= 342 non-S/R RCC); OncoPanel (N= 79 S/R and N= 395 non-S/R RCC); TCGA (N= 60 S/R and N= 828 non-S/R RCC). For all boxplots, the center of the box represents the median. The upper and lower hinges represent the 75th and 25th percentiles, respectively. The whiskers extend in both directions until the largest or lowest value not further than 1.5 times the interquartile range from the corresponding hinge. Muts: Mutations; Mb: Megabase; S/R: Sarcomatoid/Rhabdoid; TMB: Tumor Mutational Burden.

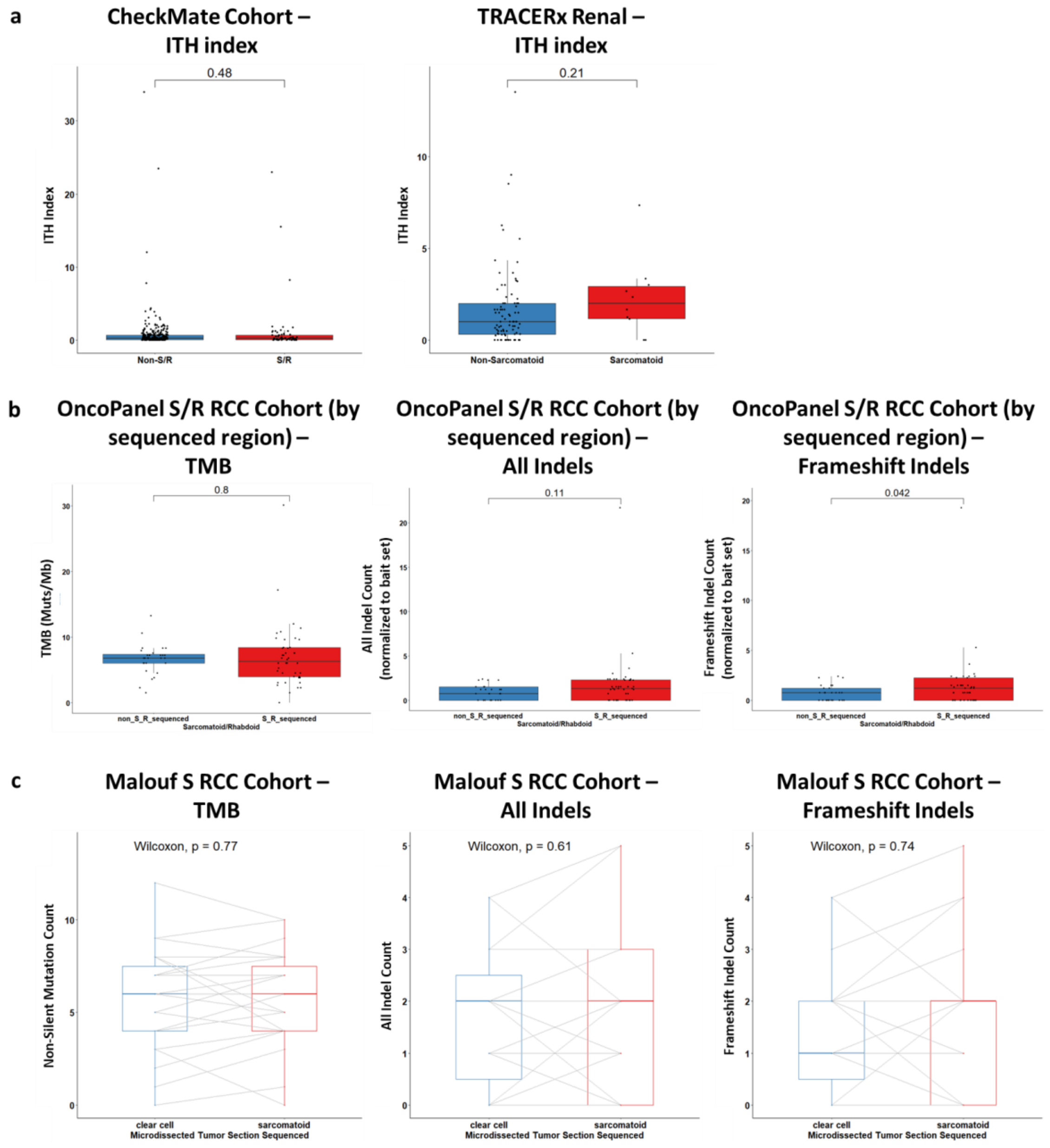

Figure S3: Limited intra-tumoral mutational heterogeneity of S/R RCC tumors (in relation to Fig. 1). (a) S/R RCC tumors have a similar intra-tumoral heterogeneity index to non-S/R RCC tumors in the CheckMate (N= 63 S/R and N= 312 non-S/R RCC) and TRACERx Renal (N= 10 S and N= 86 non-S RCC) cohorts. (b) Similar tumor mutational burden, total indel load, and frameshift indel load between the mesenchymal (S/R; N=44) and epithelioid (non-S/R; N=27) components within S/R RCC tumors in the OncoPanel cohort. (c) Similar tumor mutational burden, total indel load, and frameshift indel load between the mesenchymal (S; N=23) and epithelioid or clear cell (non-S; N=23) components within S RCC tumors in the Malouf cohort. Two-sided Mann-Whitney U test p-values shown in (a) and (b). Two-sided paired Wilcoxon signed rank test p-value shown in (c). For all boxplots, the center of the box represents the median. The upper and lower hinges represent the 75th and 25th percentiles, respectively. The whiskers extend in both directions until the largest or lowest value not further than 1.5 times the interquartile range from the corresponding hinge. Muts: Mutations; Mb: Megabase; S/R: Sarcomatoid/Rhabdoid; TMB: Tumor Mutational Burden.

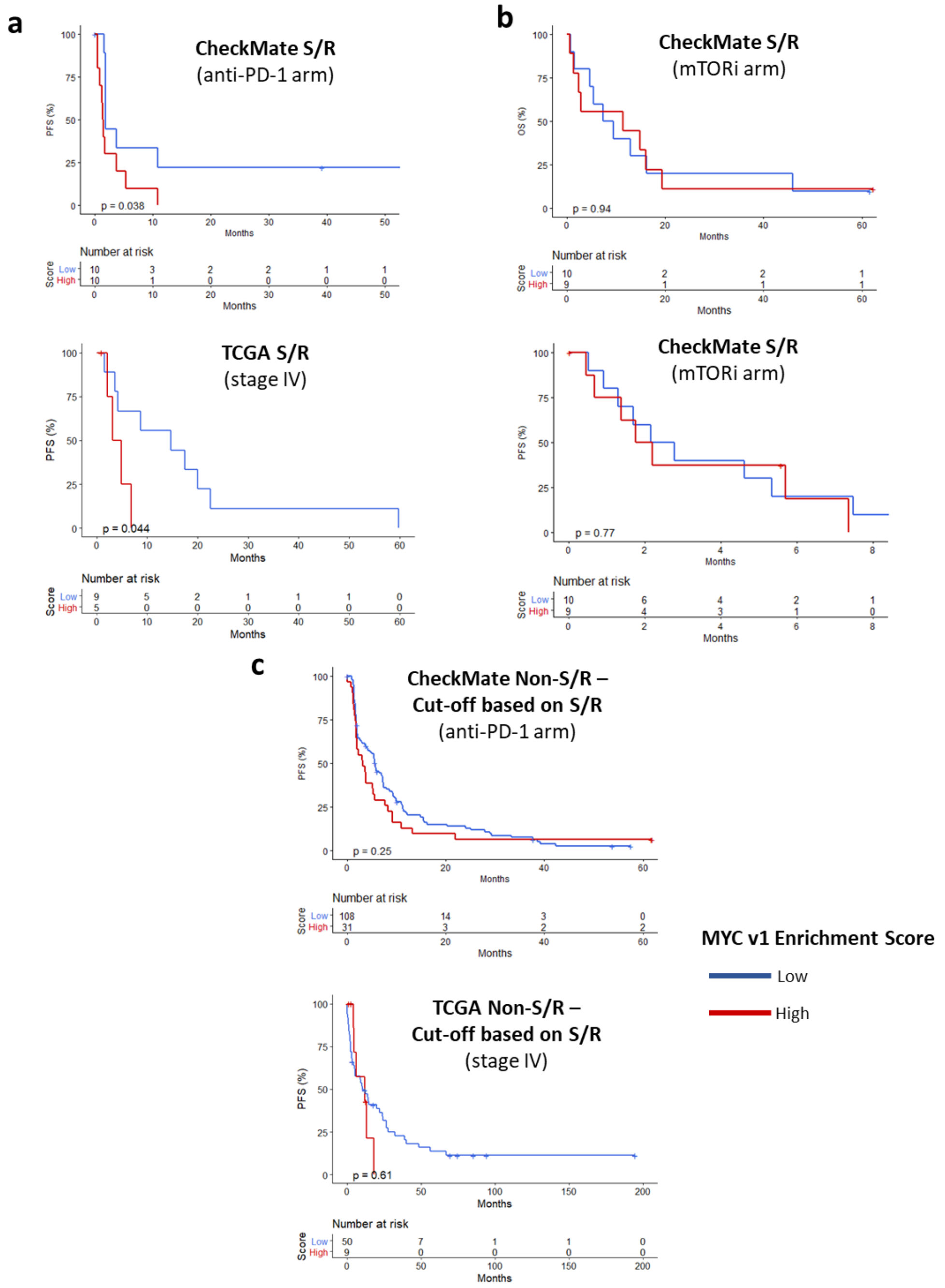

Figure S4: Transcriptional profiling of S/R RCC reveals the molecular correlates of its poor prognosis and identifies subsets of non-S/R tumors associated with a poor prognosis (in relation to Fig. 2). (a) Kaplan-Meier curves for PFS by *MYC* v1 score within the S/R group of the CheckMate (anti-PD-1 arm) and TCGA (stage IV) cohorts; *MYC* v1 score dichotomized at the median. (b) Kaplan-Meier curves for OS and PFS by *MYC* v1 score within the S/R group of the CheckMate (mTORi arm) cohort; *MYC* v1 score dichotomized at the median. (c) Kaplan-Meier curves for PFS by *MYC* v1 score within the non-S/R group of the CheckMate (anti-PD-1 arm) and TCGA (stage IV) cohorts; *MYC* v1 score dichotomized at the median of the S/R group. Log-rank test two-sided p-value reported without adjustment for multiple testing for all comparisons. *MYC* v1: *MYC* Targets Version 1; S/R: Sarcomatoid/Rhabdoid; TCGA: The Cancer Genome Atlas; mTORi: Mammalian Target of Rapamycin Inhibitors.

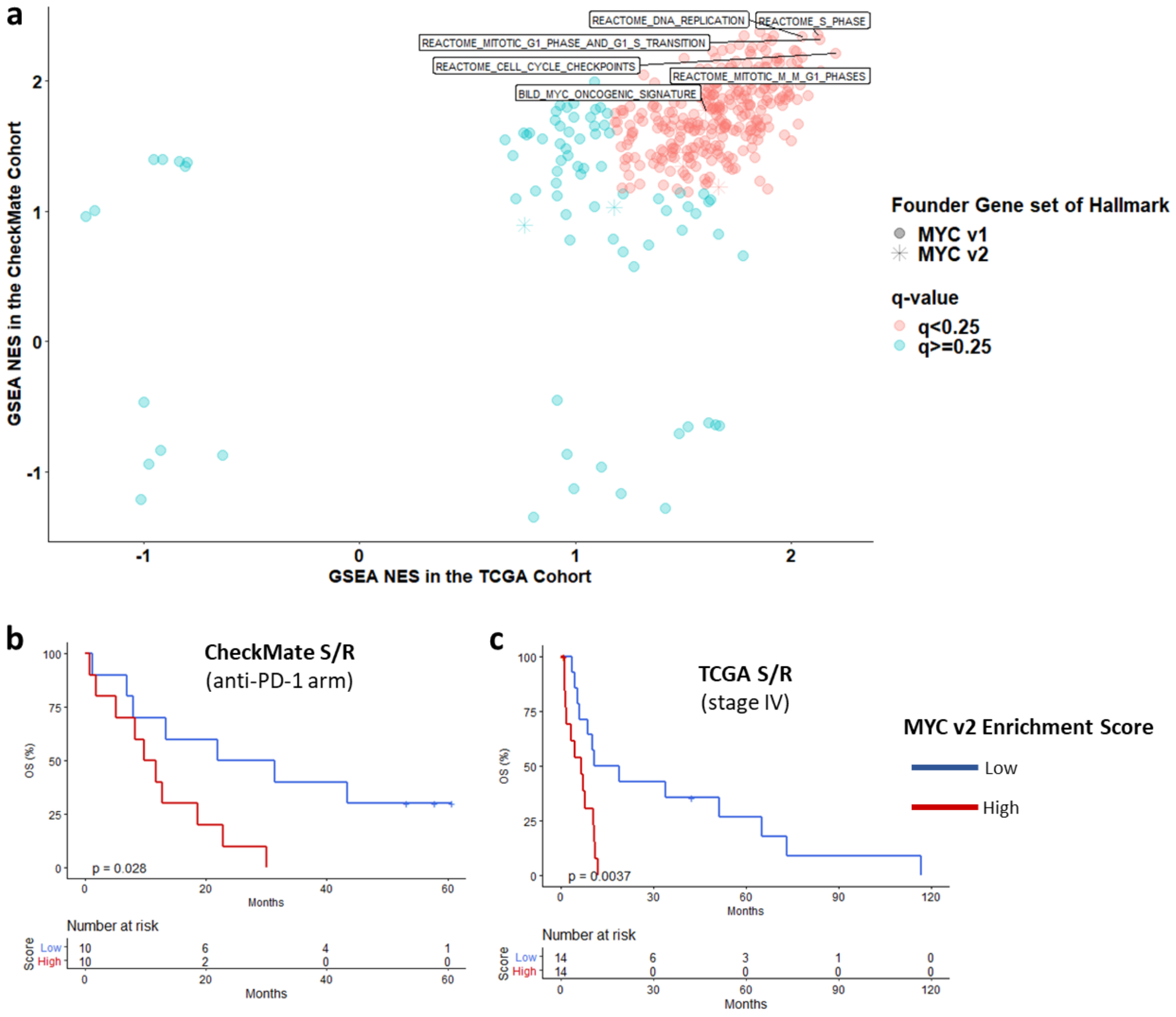

Figure S5: Upregulation of MYC-regulated gene expression and correlation with outcomes in S/R RCC (in relation to Fig. 2). (a) Enrichment of “Founder” gene sets of the “Hallmark” *MYC* v1 and v2 gene sets in the CheckMate and TCGA cohorts by GSEA. P-value calculated using a phenotype permutation-based two-sided test with 1000 permutations. Adjustments for multiple testing were made using the false discovery rate (FDR) method. (b) Kaplan-Meier curves for OS by *MYC* v2 score within the S/R group of the CheckMate (anti-PD-1 arm) and TCGA (stage IV) cohorts; *MYC* v1 score dichotomized at the median. Log-rank test two-sided p-value reported without adjustment for multiple testing. GSEA: Gene Set Enrichment Analysis; *MYC* v2: *MYC* Targets Version 2; NES: Normalized Enrichment Score; S/R: Sarcomatoid/Rhabdoid; TCGA: The Cancer Genome Atlas.

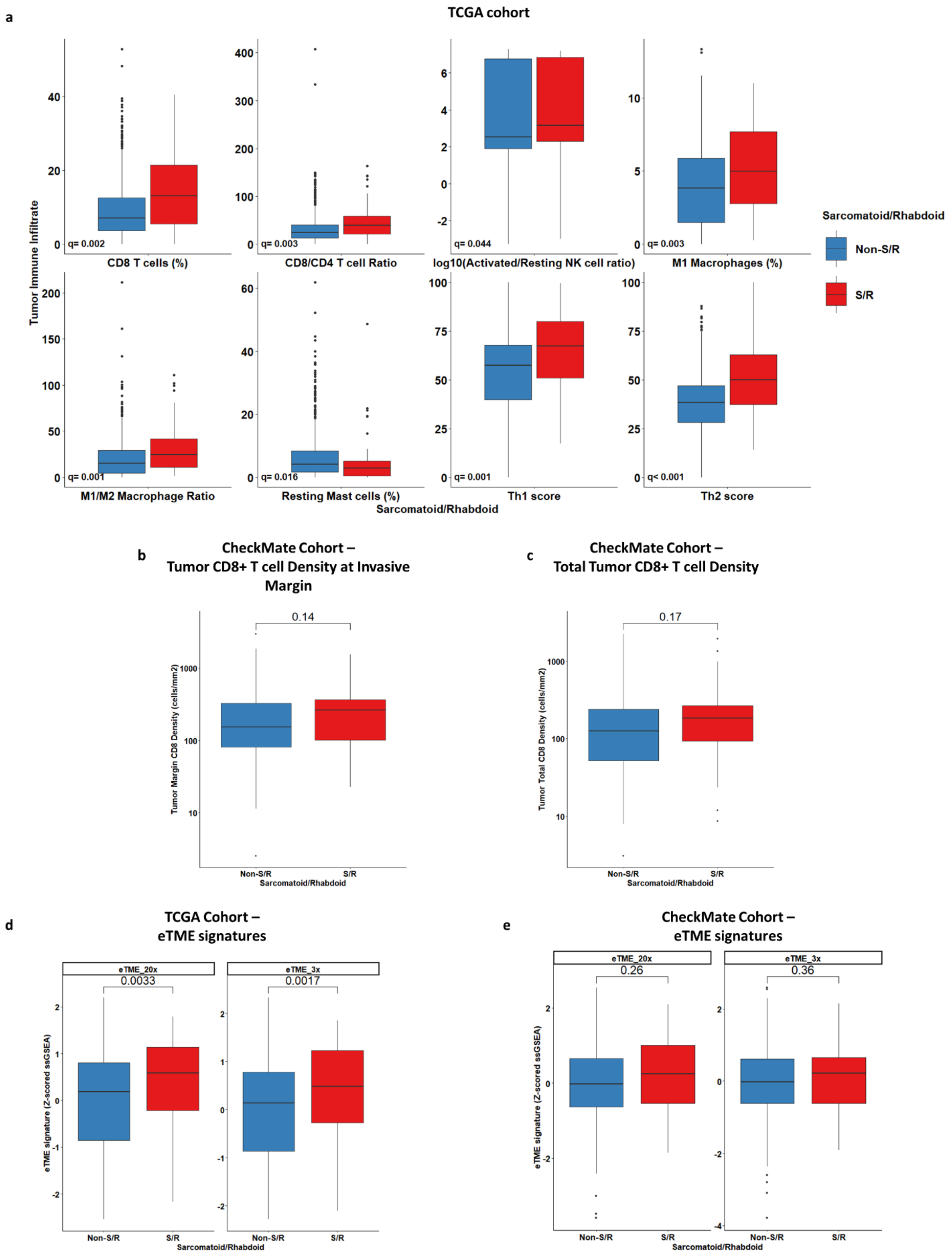

Figure S6: The improved outcomes of S/R RCC tumors on immune checkpoint inhibitors across clinical trial and real-word cohorts may be accounted for by an immune-inflamed phenotype (in relation to Fig. 4). (a) Boxplots of the comparison of CIBERSORTx and T helper immune cell populations between S/R (N= 58) and non-S/R RCC (N= 782), with two-sided Mann-Whitney U test comparisons corrected for multiple comparison testing (q value reported). Only

variables which were significant (q<0.05) in both the CheckMate and TCGA cohorts independently were shown. The TCGA results are displayed in this figure. Boxplots of the comparison of CD8+ T cell density at the (b) tumoral invasive margin and (c) throughout the tumor as determined by immunofluorescent staining in S/R (N= 29) compared to non-S/R RCC (N= 186). Two-sided Mann-Whitney U test p-values reported. Boxplots of the comparison of Z-scored ssGSEA scores of the empirical tumor microenvironment signatures (eTME) at 20-fold and 3-fold cut-offs between S/R and non-S/R RCC in the (d) TCGA (S/R N= 59 and non-S/R N= 830) and (e) CheckMate (S/R N= 39 and non-S/R N= 247) cohorts. Two-sided Mann-Whitney U test p-values reported. For all boxplots, the center of the box represents the median. The upper and lower hinges represent the 75th and 25th percentiles, respectively. The whiskers extend in both directions until the largest or lowest value not further than 1.5 times the interquartile range from the corresponding hinge. Outliers (beyond 1.5 times the interquartile range) are plotted individually. S/R: Sarcomatoid/Rhabdoid; TCGA: The Cancer Genome Atlas; ssGSEA: Single Sample Gene Set Enrichment Analysis.

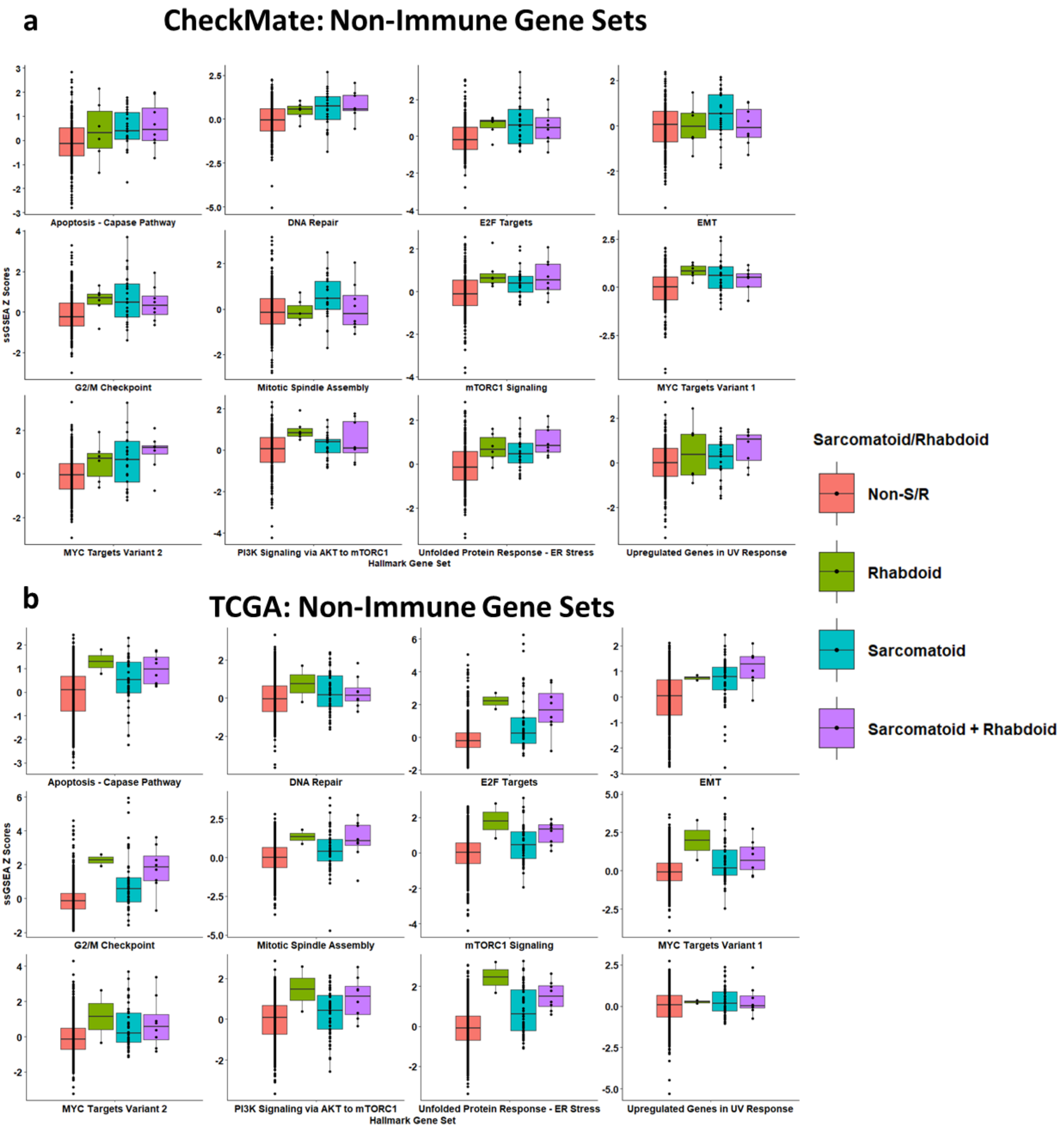

Figure S7: Transcriptomic programs of sarcomatoid, rhabdoid, and sarcomatoid and rhabdoid tumors. Breakdown of Z-score normalized ssGSEA scores in non-sarcomatoid/rhabdoid, sarcomatoid, rhabdoid, and sarcomatoid and rhabdoid tumors of significantly enriched non-immune GSEA pathways in S/R RCC in the (a) CheckMate (non-sarcomatoid/rhabdoid N= 247, sarcomatoid N= 25, rhabdoid N= 6, and sarcomatoid and rhabdoid N= 8) and (b) TCGA cohorts (non-sarcomatoid/rhabdoid N= 830, sarcomatoid N= 49, rhabdoid N= 2, and sarcomatoid and rhabdoid N= 8; in relation to Fig. 2). For all boxplots, the center of the box represents the median. The upper and lower hinges represent the 75th and 25th percentiles, respectively. The whiskers extend in both directions until the largest or lowest value not further than 1.5 times the interquartile range from the corresponding hinge. EMT: Epithelial Mesenchymal Transition; S/R: Sarcomatoid/Rhabdoid; ssGSEA: Single Sample Gene Set Enrichment Analysis.

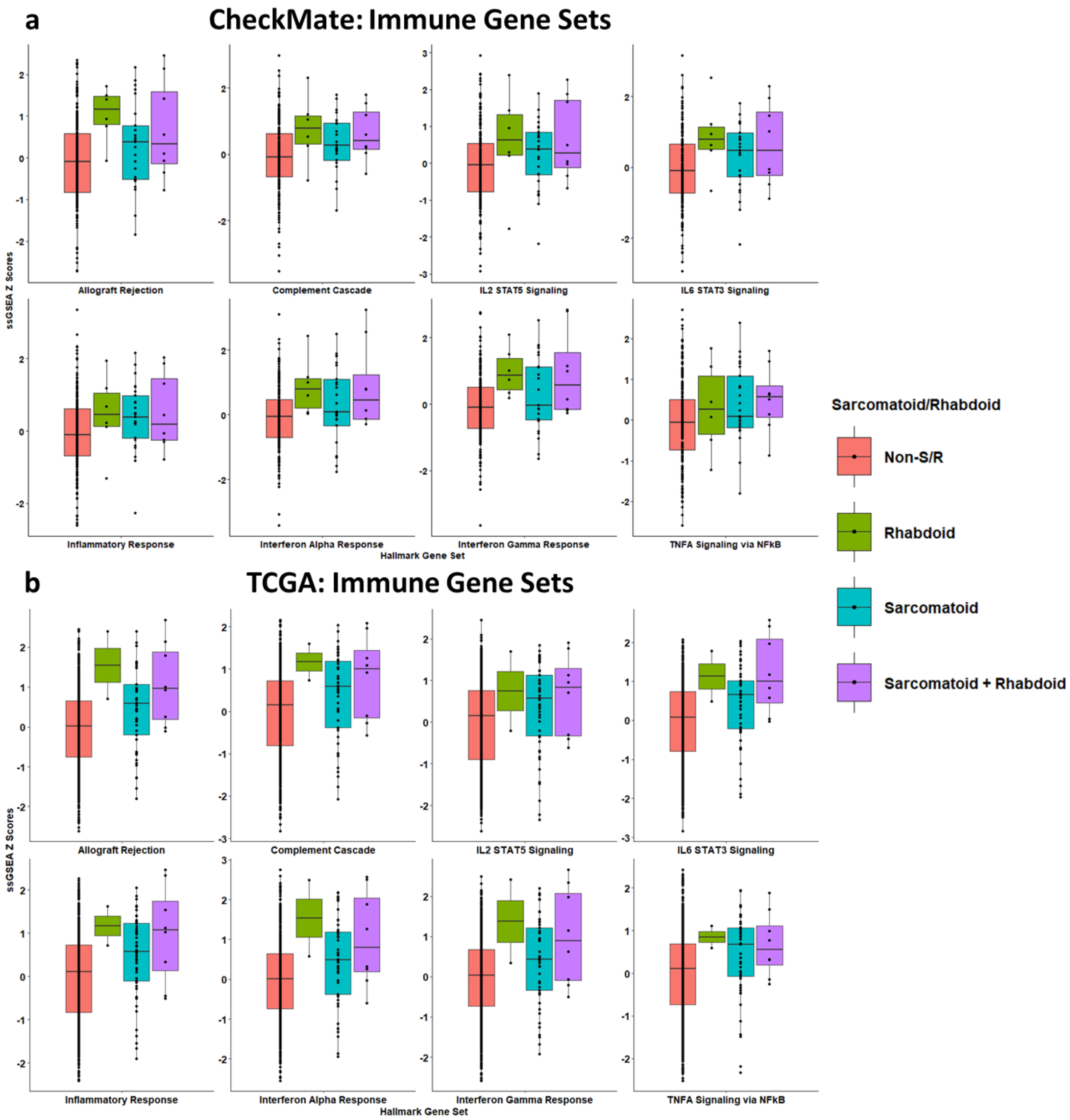

Figure S8: Immune transcriptomic programs of sarcomatoid, rhabdoid, and sarcomatoid and rhabdoid tumors. Breakdown of Z-score normalized ssGSEA scores in non-sarcomatoid/rhabdoid, sarcomatoid, rhabdoid, and sarcomatoid and rhabdoid tumors of significantly enriched immune GSEA pathways in S/R RCC in the (a) CheckMate (non-sarcomatoid/rhabdoid N= 247, sarcomatoid N= 25, rhabdoid N= 6, and sarcomatoid and rhabdoid N= 8) and (b) TCGA cohorts (non-sarcomatoid/rhabdoid N= 830, sarcomatoid N= 49, rhabdoid N= 2, and sarcomatoid and rhabdoid N= 8; in relation to Fig. 4). For all boxplots, the center of the box represents the median. The upper and lower hinges represent the 75th and 25th percentiles, respectively. The whiskers extend in both directions until the largest or lowest value not further than 1.5 times the interquartile range from the corresponding hinge. S/R: Sarcomatoid/Rhabdoid; ssGSEA: Single Sample Gene Set Enrichment Analysis.

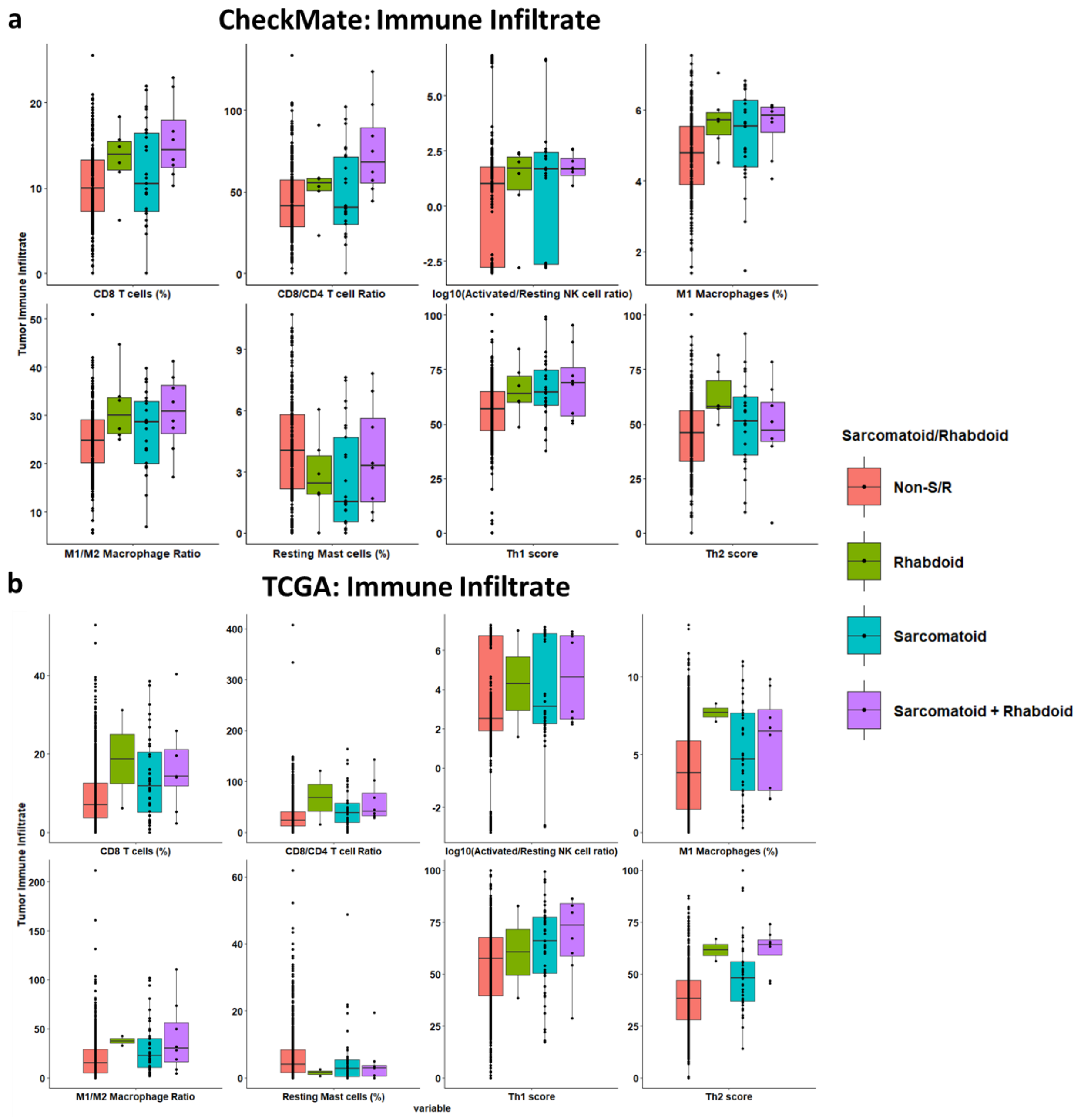

Figure S9: CIBERSORTx-inferred immune infiltrates of sarcomatoid, rhabdoid, and sarcomatoid and rhabdoid tumors. Breakdown of differentially enriched infiltrating immune cell populations in non-sarcomatoid/rhabdoid, sarcomatoid, rhabdoid, and sarcomatoid and rhabdoid tumors in the (a) CheckMate (non-sarcomatoid/rhabdoid N= 247, sarcomatoid N= 25, rhabdoid N= 6, and sarcomatoid and rhabdoid N= 8) and (b) TCGA (non-sarcomatoid/rhabdoid N= 782, sarcomatoid N= 48, rhabdoid N= 2, and sarcomatoid and rhabdoid N= 8) cohorts (in relation to Fig. 4). For all boxplots, the center of the box represents the median. The upper and lower hinges represent the 75th and 25th percentiles, respectively. The whiskers extend in both directions until the largest or lowest value not further than 1.5 times the interquartile range from the corresponding hinge. S/R: Sarcomatoid/Rhabdoid.

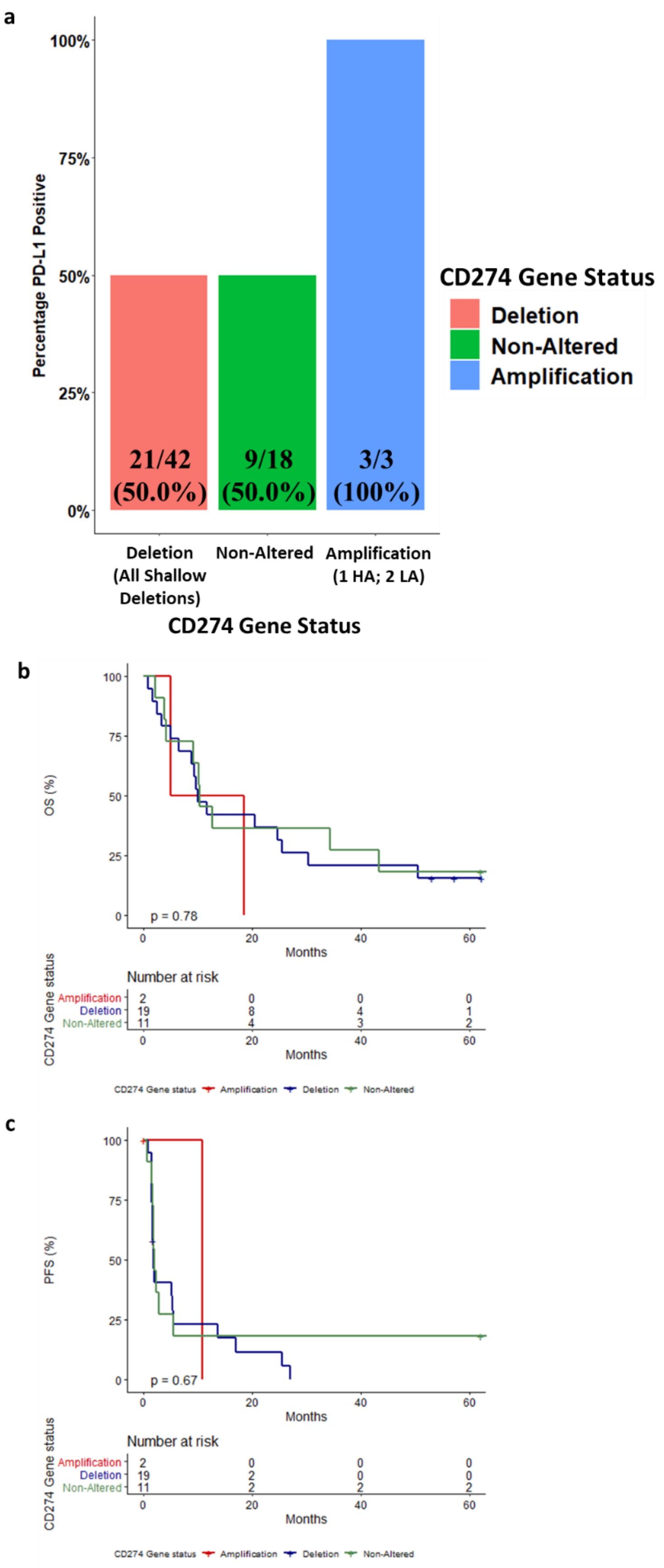

Figure S10: The improved outcomes of S/R RCC tumors on immune checkpoint inhibitors are not accounted for by *CD274* gene amplification. (a)

Relationship between *CD274* (or PD-L1) gene status and PD-L1 expression in the subgroup of patients with S/R RCC that had WES and PD-L1 expression evaluated by IHC. Relationship between *CD274* (or PD-L1) gene status and survival outcomes on nivolumab in the subgroup of patients with S/R RCC that had WES and were treated by nivolumab; (b) OS and (c) PFS (in relation to Fig. 4). HA: High Amplification; LA: Low Amplification; OS: Overall Survival; PFS: Progression Free Survival.

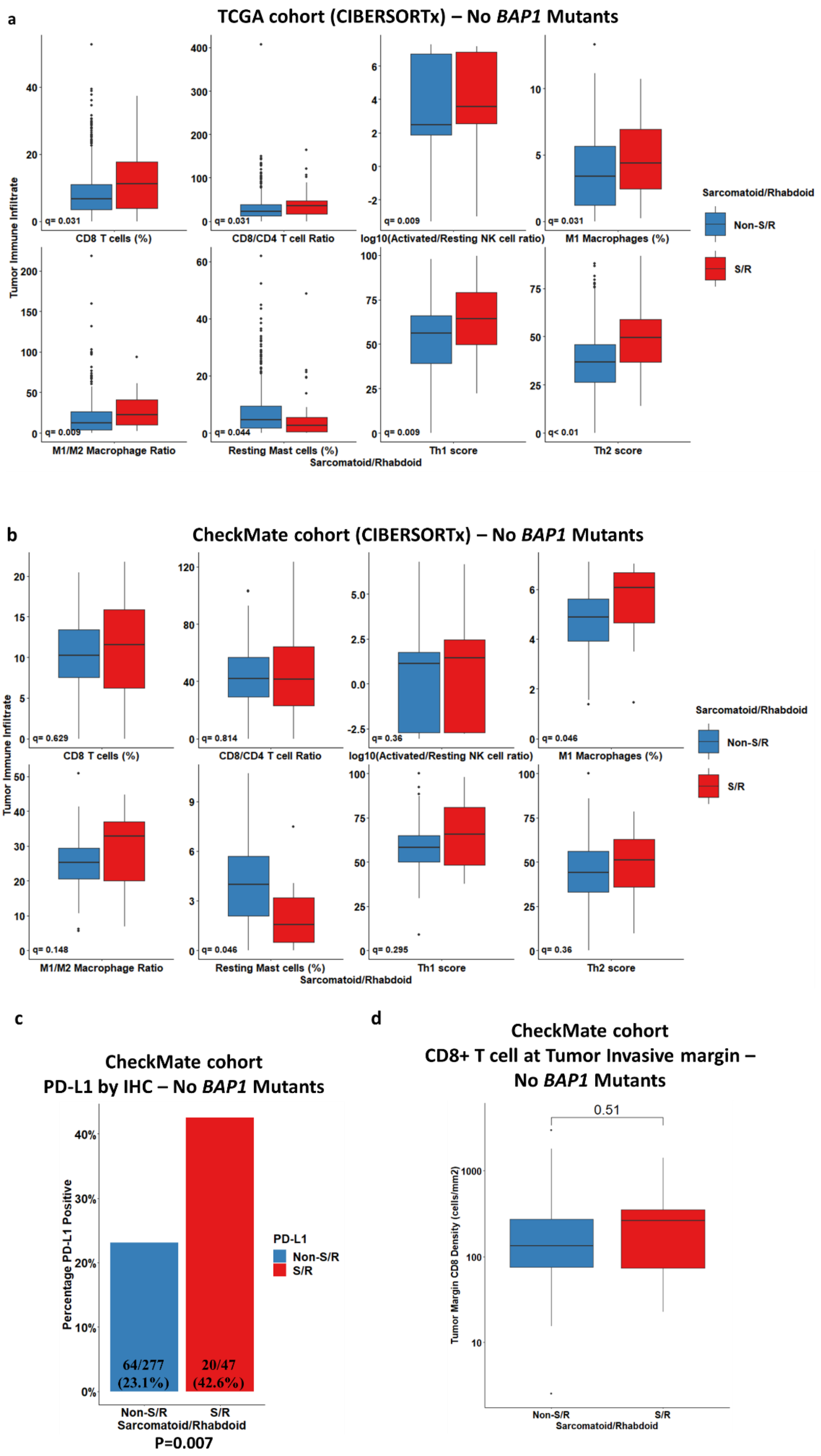

Figure S11: The immune-inflamed phenotype of S/R RCC tumors is independent of *BAP1* mutations. All plots exclude tumors with *BAP1* mutations in

both the S/R and non-S/R RCC groups (in relation to Fig. 4). Boxplots of the comparison of CIBERSORTx and T helper immune cell populations between S/R and non-S/R RCC, with two-sided Mann-Whitney U test (q-value reported) in the (a) TCGA (N= 53 S/R and N= 593 non-S/R RCC) and (b) CheckMate cohorts, excluding *BAP1* mutants (N= 13 S/R and N= 146 non-S/R RCC). (c) Bar plot of the comparison of the proportions of tumors that were PD-L1 positive (≥1% on tumor cells) in S/R compared to non-S/R RCC, excluding *BAP1* mutants. Two-sided Fisher's exact test p-value reported. (d) Boxplot of the comparison of CD8+ T cell density at the tumoral invasive margin between S/R and non-S/R RCC, excluding *BAP1* mutants (N= 15 S/R and N= 99 non-S/R RCC). Two-sided Mann-Whitney U test p-value reported. For all boxplots, the center of the box represents the median. The upper and lower hinges represent the 75th and 25th percentiles, respectively. The whiskers extend in both directions until the largest or lowest value not further than 1.5 times the interquartile range from the corresponding hinge. Outliers (beyond 1.5 times the interquartile range) are plotted individually. TCGA: The Cancer Genome Atlas.

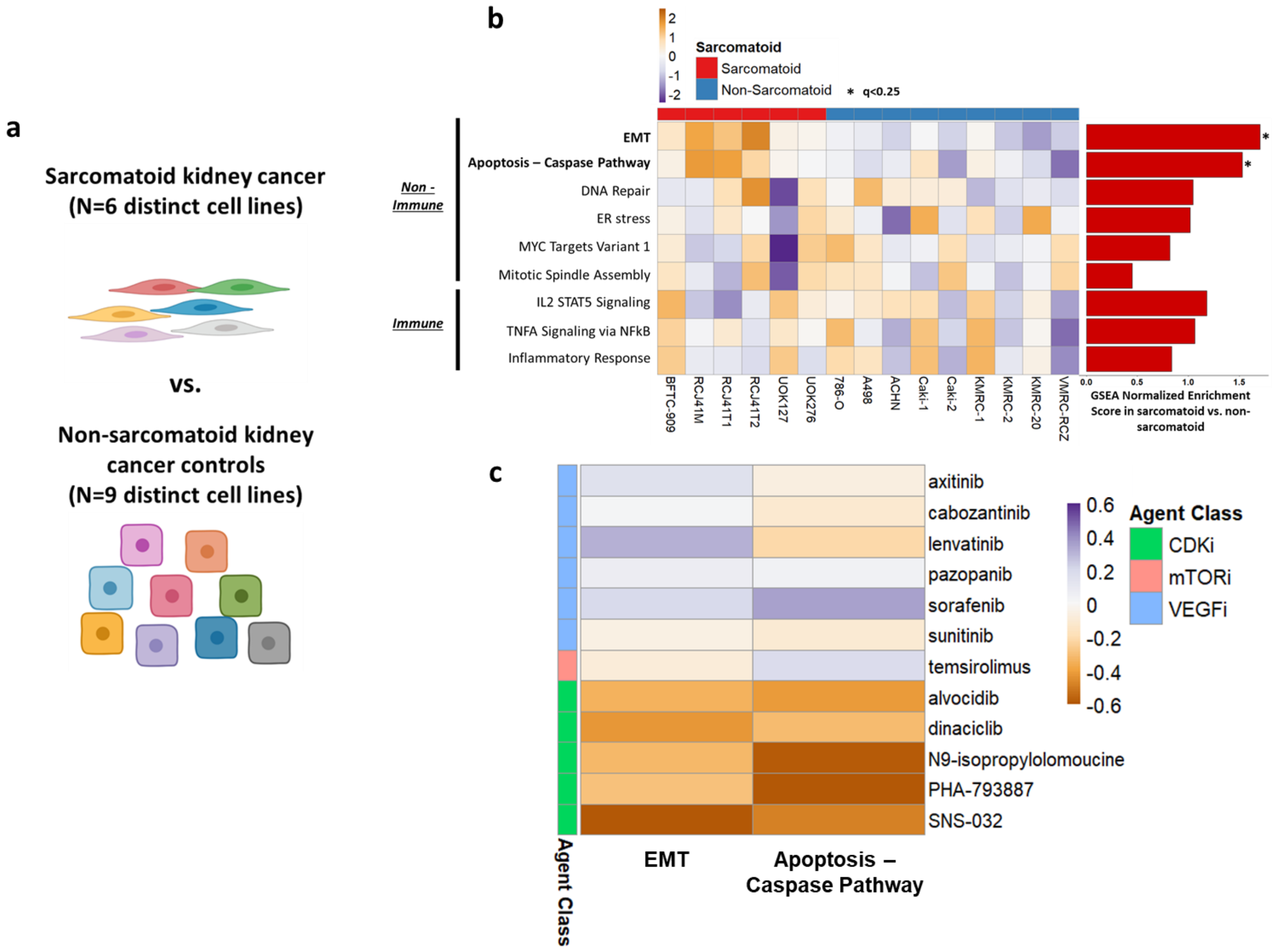

Figure S12: Baseline transcriptomic profiling of kidney cancer cell lines reveals that both immune and non-immune features of sarcomatoid tumors may be driven by the sarcomatoid component. (a) GSEA was performed on the 50 "Hallmark" gene sets to compare 6 distinct sarcomatoid cell lines and 9 distinct non-sarcomatoid kidney cancer cell lines. (b) Heatmap and bar plot of the ssGSEA scores and GSEA normalized enrichment scores for the "Hallmark" gene sets that were found to be enriched in sarcomatoid compared to non-sarcomatoid cell lines. P-value calculated using a phenotype permutation-based two-sided test with 1000 permutations. Adjustments for multiple testing (50 "Hallmark" gene sets) were made using the false discovery rate (FDR) method. (c) Heatmap of the Pearson correlation coefficients between the area under curve (AUC) of the dose-response curve and the ssGSEA scores of the two pathways which were found to be significantly enriched in both cohorts of bulk RNA-seq and in the sarcomatoid cell lines (epithelial-mesenchymal transition and the apoptosis-caspase pathway). Agents are grouped by drug class and the color orange in this heatmap represents a negative correlation between ssGSEA score and AUC (indicating that a higher ssGSEA score correlates with greater drug sensitivity). The agents included in this figure are CDKi as well as the mTORi and VEGFi that are FDA-approved for metastatic renal cell carcinoma (for comparison). *q<0.25; CDKi: Cyclin-Dependent-Kinase Inhibitors; EMT: Epithelial Mesenchymal Transition; FDA: Food and Drug Administration; mTORi: Mammalian Target of Rapamycin Inhibitors; VEGFi: Vascular Endothelial Growth Factor Inhibitors.

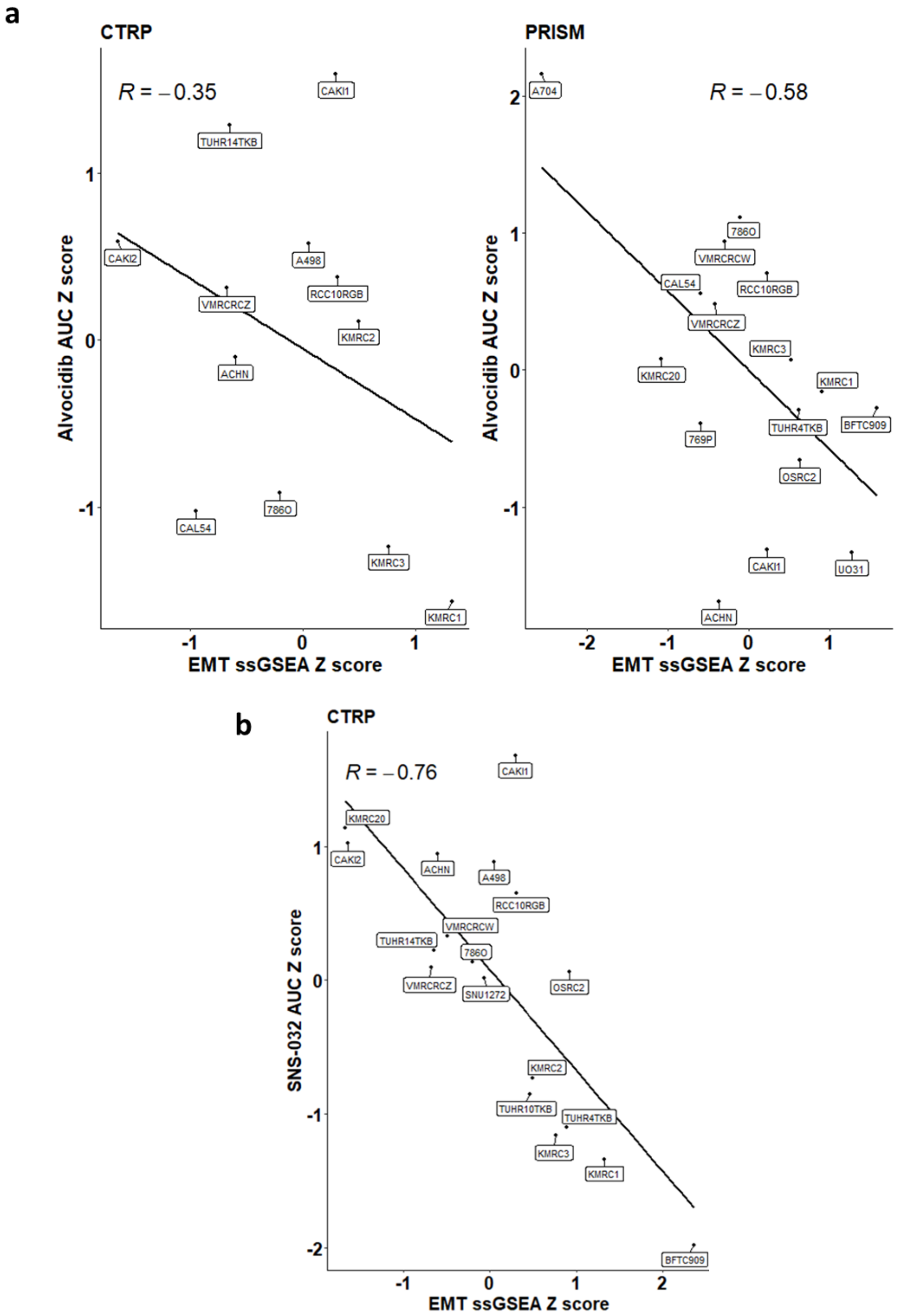

Figure S13: Scatter plots of correlations of transcriptomic characteristics of cell lines with areas under the curve of dose response curves in CTRP and PRISM for two CDK inhibitors. (a) alvocidib and (b) SNS-032. Pearson r correlation coefficients shown. AUC: Area Under the Curve; EMT: Epithelial Mesenchymal Transition.

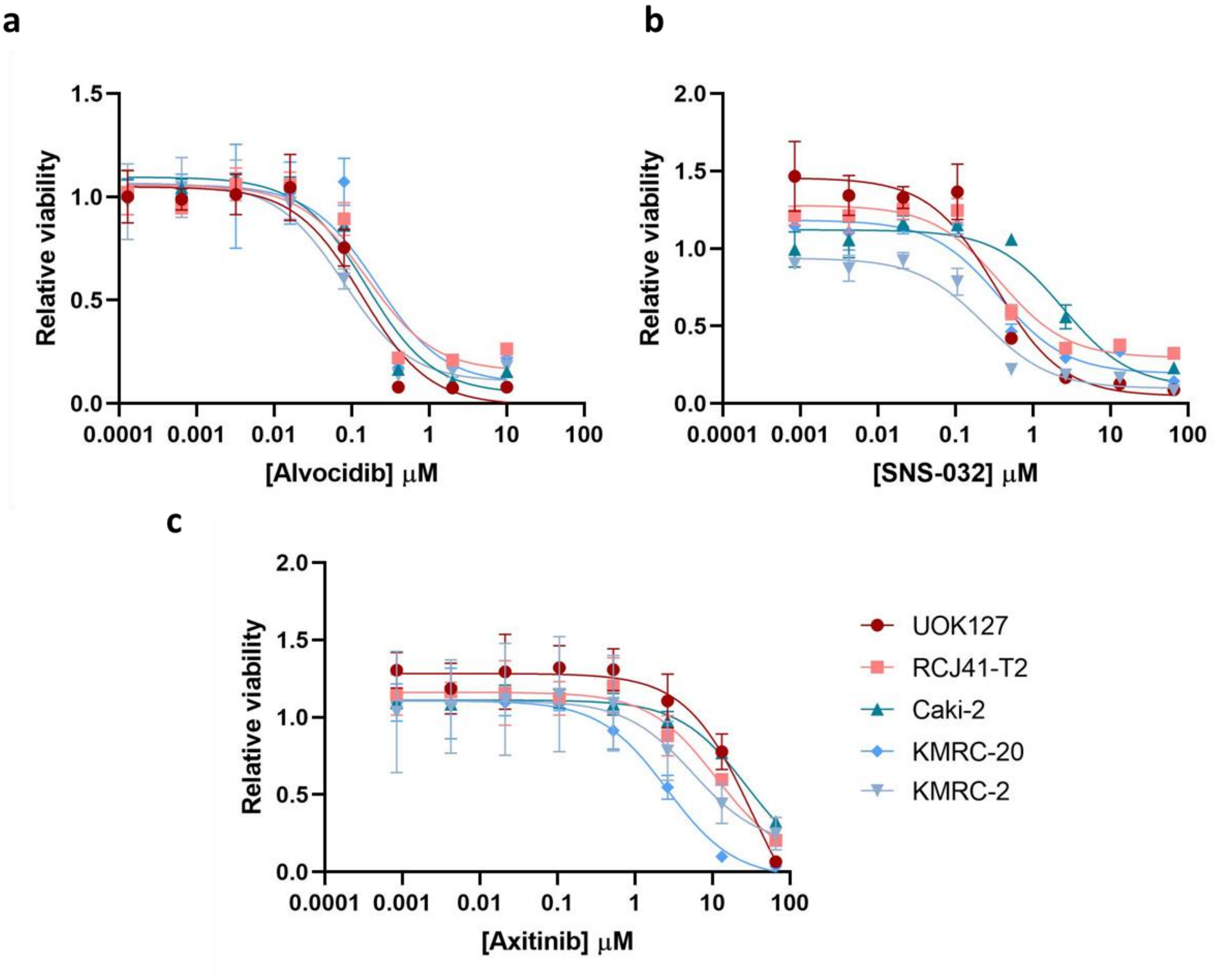

Figure S14: Selected drug sensitivity profiles of sarcomatoid and non-sarcomatoid cell line models. Dose-response curves of the in vitro cell line drug sensitivity assays for (a) alvocidib, (b) SNS-032, and (c) axitinib in two sarcomatoid cell lines (UOK 127 and RCJ41-T2) and three non-sarcomatoid cell lines (Caki-2, KMRC-20, KMRC-2). N= 4 biological replicates of each cell line under each treatment condition. Data are presented as mean +/- 1 standard deviation.

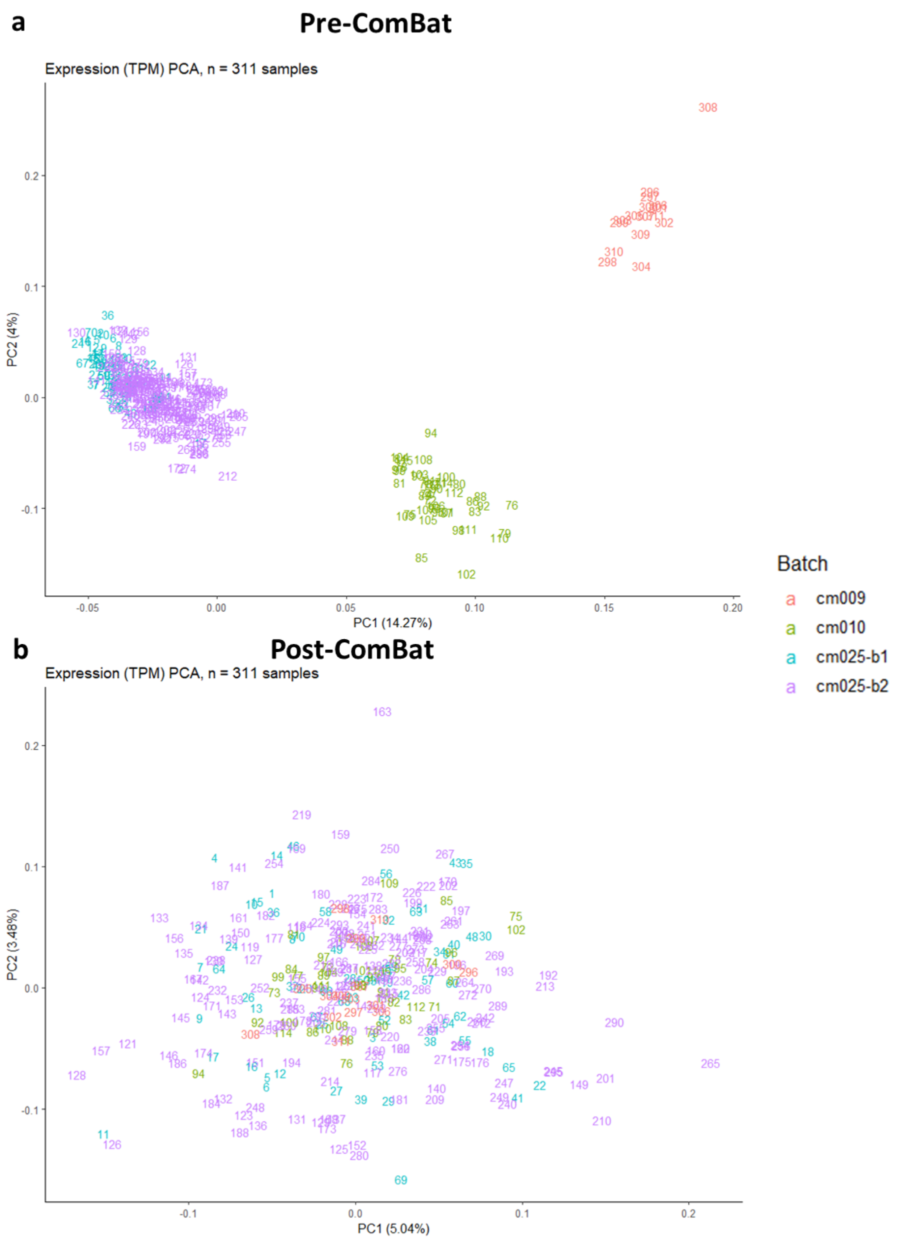

Figure S15: RNA-sequencing batch effect correction. Principal component analysis plots of the UQ-normalized log2-transformed TPM matrix including the 3 known batches within the CheckMate cohort (a) pre-ComBat and (b) post-ComBat. cm010: CheckMate 010; cm-025-b1: CheckMate 025 Batch 1; cm-025-b2: CheckMate 025 Batch 2; PC1: Principal Component 1; PC2: Principal Component 2; PCA : Principal Component Analysis ; TPM : Transcripts-Per-Million.
