# Supplementary material for: Integrative molecular characterization of sarcomatoid and rhabdoid renal cell carcinoma
Source: Nat Commun. 2021 Feb 5;12:808. doi: 10.1038/s41467-021-21068-9 (PMC7865061; doi:10.1038/s41467-021-21068-9)
Supplement: Supplementary file 3 — Description of Additional Supplementary Files [file 41467_2021_21068_MOESM3_ESM.pdf]

Description of Additional Supplementary Files

Dataset 1: Baseline characteristics of the TCGA, CheckMate, and OncoPanel genomic cohorts, and clinical and genomic data of the OncoPanel cohort.

Dataset 2: Genomic analysis results of the TCGA, CheckMate, and OncoPanel genomic cohorts, genomic meta-analysis results, and breakdown of genomic alterations by background histology in the TCGA and OncoPanel cohorts. P-values reported within each dataset for two-sided Fisher's exact test with Fisher's meta-analysis method used to combine p-values. The combined p-values were corrected for multiple hypothesis testing using Benjamini-Hochberg correction (q-value reported).

Dataset 3: Gene level enrichment analyses of mutations in the OncoPanel cohort between epithelioid and S/R components of different S/R RCC tumors (two-sided Fisher's exact tests) and in the Malouf cohort between epithelioid and S components of the same S RCC tumors (two-sided McNemar tests). P-values were corrected for multiple hypothesis testing using Benjamini-Hochberg correction (q-value reported).

Dataset 4: Baseline characteristics of the TCGA and CheckMate RNA-sequencing cohorts.

Dataset 5: "Hallmark" and antigen presentation machinery gene set enrichment analysis results in the TCGA and CheckMate RNA-sequencing cohorts. P-value calculated using a phenotype permutation-based two-sided test with 1000 permutations. Adjustments for multiple testing were made using the false discovery rate (FDR) method. A separate procedure for multiple testing correction is also reported (familywise-error rate [FWER]).

Dataset 6: "Hallmark" single sample gene set enrichment analysis in the TCGA and CheckMate RNA-sequencing cohorts and results of Cox regression analysis with overall survival. Univariable two-sided p-values from Cox regression reported. P-values were corrected for multiple hypothesis testing using Benjamini-Hochberg correction (q-value reported).

Dataset 7: Gene-level differential gene expression analysis results (two-sided Mann-Whitney U test results) with log2 fold-changes of the mean. p-values were corrected for multiple hypothesis testing using Benjamini-Hochberg correction (q-value reported). Genes that are significantly ($q<0.05$) upregulated or downregulated in the TCGA and CheckMate cohorts independently are also highlighted in separate tabs.

Dataset 8: Baseline characteristics of the Harvard, IMDC, and CheckMate clinical cohorts.

Dataset 9: CIBERSORTx deconvolution results in absolute mode of the CheckMate and TCGA cohorts with single sample gene set enrichment scores for Th1, Th2, and Th17 cells (scaled between 0 and 100) and two-sided Mann-Whitney U test comparison results in the TCGA and CheckMate cohorts independently. p-values were corrected for multiple hypothesis testing using Benjamini-Hochberg correction (q-value reported).

Dataset 10: Baseline characteristics of patients that had their tumor tissue stained by immunohistochemistry for PD-L1 or CD8+ T cells by immunofluorescence.

Dataset 11: Raw and transformed TPM matrix of the 15 sequenced cell lines, quality control metrics by RNA-seqQC2, "Hallmark" gene set enrichment analysis of sarcomatoid vs. non-sarcomatoid cell lines, "Hallmark" single-sample gene set enrichment analysis of all 15 cell lines, epithelial-mesenchymal transition and apoptosis-caspase pathway single-sample gene set enrichment analysis of the 20 kidney cancer cell lines in CTRP v2 with drug sensitivity data, Pearson r correlation coefficients between single sample gene set enrichment analysis scores and areas under the curve (AUC) of the dose-response curves for the 20 kidney cancer cell lines in CTRP v2 and in the PRISM secondary screen. For GSEA, the p-value was calculated using a phenotype permutation-based two-sided test with 1000 permutations. Adjustments for multiple testing were made using the false discovery rate (FDR) method. A separate procedure for multiple testing correction is also reported (familywise-error rate [FWER]).

Dataset 12: Sarcomatoid and rhabdoid annotation for the TCGA KIPAN cohort.

Dataset 13: List of genes evaluated in the genomic analysis and table indicating which genes were included in each version of the OncoPanel assay.

Dataset 14: Patient-level clinical data of the Harvard cohort.
