# Supplementary material for: Integrative molecular characterization of sarcomatoid and rhabdoid renal cell carcinoma
Source: Nat Commun. 2021 Feb 5;12:808. doi: 10.1038/s41467-021-21068-9 (PMC7865061; doi:10.1038/s41467-021-21068-9)
Supplement: Supplementary file 18 — Reporting Summary [file 41467_2021_21068_MOESM18_ESM.pdf]

nature research

Corresponding author(s): Eliezer M. Van Allen and Toni K. Choueiri

Last updated by author(s): 12/14/2020

Reporting Summary

Nature Research wishes to improve the reproducibility of the work that we publish. This form provides structure for consistency and transparency in reporting. For further information on Nature Research policies, see Authors & Referees and the Editorial Policy Checklist.

Statistics

For all statistical analyses, confirm that the following items are present in the figure legend, table legend, main text, or Methods section.

n/a	Confirmed	
☐	☒	The exact sample size (*n*) for each experimental group/condition, given as a discrete number and unit of measurement
☐	☒	A statement on whether measurements were taken from distinct samples or whether the same sample was measured repeatedly
☐	☒	The statistical test(s) used AND whether they are one- or two-sided<br>*Only common tests should be described solely by name; describe more complex techniques in the Methods section.*
☒	☐	A description of all covariates tested
☐	☒	A description of any assumptions or corrections, such as tests of normality and adjustment for multiple comparisons
☐	☒	A full description of the statistical parameters including central tendency (e.g. means) or other basic estimates (e.g. regression coefficient) AND variation (e.g. standard deviation) or associated estimates of uncertainty (e.g. confidence intervals)
☐	☒	For null hypothesis testing, the test statistic (e.g. *F*, *t*, *r*) with confidence intervals, effect sizes, degrees of freedom and *P* value noted<br>*Give P values as exact values whenever suitable.*
☒	☐	For Bayesian analysis, information on the choice of priors and Markov chain Monte Carlo settings
☒	☐	For hierarchical and complex designs, identification of the appropriate level for tests and full reporting of outcomes
☐	☒	Estimates of effect sizes (e.g. Cohen's *d*, Pearson's *r*), indicating how they were calculated

Our web collection on statistics for biologists contains articles on many of the points above.

Software and code

Policy information about availability of computer code

Data collection	HALO Image Analysis platform version 2.1.1637.18 was used for immunofluorescence image analysis.
Data analysis	Statistical analyses was done in R Statistical software (version 3.6.1). GraphPad PRISM 8 for cell line in vitro assay data analysis. The Broad Institute's Desktop Java Application v4.0.0 was used for gene set enrichment analysis (GSEA).

For manuscripts utilizing custom algorithms or software that are central to the research but not yet described in published literature, software must be made available to editors/reviewers. We strongly encourage code deposition in a community repository (e.g. GitHub). See the Nature Research guidelines for submitting code & software for further information.

Data

Policy information about availability of data

All manuscripts must include a data availability statement. This statement should provide the following information, where applicable:

- Accession codes, unique identifiers, or web links for publicly available datasets
- A list of figures that have associated raw data
- A description of any restrictions on data availability

All relevant data are available from the authors and/or are included with the manuscript. All clinical and correlative data from the CheckMate 010 and 025 clinical trials are made separately available as part of the accompanying paper50. WES data from the CheckMate 010 and 025 clinical trials from patients who consented to deposition have been submitted to the European Genome-phenome Archive (Accession numbers EGAS00001004291 and EGAS00001004292). All intermediate data from the RNA-seq analyses of the CheckMate and TCGA cohorts are made available in tables S6 (single sample gene set enrichment analysis scores) and S9 (CIBERSORTx immune deconvolution). The raw, transformed, and intermediate data from the generated cell line RNA-seq data are made available in Table S11. The clinical data from the Harvard cohort are available in Table S14. For the TCGA cohort, publicly available data was downloaded for mutation data (https://gdc.cancer.gov/about-data/publications/mc3-2017), CNA data (https://www.cbioportal.org/datasets), RNA-seq data (https://www.cbioportal.org/datasets), and clinical data (https://www.cbioportal.org/datasets). The dataset from the study by Malouf et al. of paired sequencing of sarcomatoid RCC was downloaded from https://www.nature.com/articles/s41598-020-57534-5#Sec16 (supplementary dataset 1). The dataset from the TRACERx Renal study was downloaded from https://

www.ncbi.nlm.nih.gov/pmc/articles/PMC5938372/ (Tables S1 and S2). RNA-seq data for 20 kidney cancer cell lines with RNA-seq and drug sensitivity data were downloaded from The Cancer Dependency Map Portal (DepMap) (https://depmap.org/portal/download/) and drug sensitivity data were downloaded from the Cancer Therapeutics Response Portal (CTRP v2) (https://portals.broadinstitute.org/ctrp/?cluster=true?page=#ctd2Cluster) and the PRISM 19Q4 secondary screen (https://depmap.org/portal/download/) as areas under the curve (AUC) for all agents. Exome Sequencing Project database (http://evs.gs.washington.edu/EVS/) and 1000 Genomes Project data (https://www.internationalgenome.org/data) to detect potential germline variants from tumor-only gene panel sequencing data. MSigDB 7.0 (https://www.gsea-msigdb.org/gsea/msigdb) was used to define gene pathways of interest. Any other queries about the data used in this study should be directed to the corresponding authors of this study.

Field-specific reporting

Please select the one below that is the best fit for your research. If you are not sure, read the appropriate sections before making your selection.

☒ Life sciences ☐ Behavioural & social sciences ☐ Ecological, evolutionary & environmental sciences

For a reference copy of the document with all sections, see nature.com/documents/nr-reporting-summary-flat.pdf

Life sciences study design

All studies must disclose on these points even when the disclosure is negative.

Sample size

The presented results are mainly from secondary analyses of data collected from multiple cohorts and data sources (TCGA, DF/HCC, CheckMate 010 & 025, IMDC, Malouf cohort, TRACERx Renal cohort, DepMap, CTRP v2, and PRISM). The effective sample size used in this study corresponds to the subset of samples that had not failed sequencing and that had been consented to have their samples collected for sequencing. The overall sample sizes by type of data/analysis are summarized in Figure 1a.
Overall, the effective sample sizes were 1773 patients with DNA sequencing (1565 non-S/R and 208 S/R RCC), 1175 with RNA-sequencing (1077 non-S/R and 98 S/R RCC), 1250 patients with S/R RCC with clinical outcomes on systemic therapy (1013 non-ICI treated and 237 ICItreated),
809 with IHC for PD-L1 (691 non-S/R and 118 S/R RCC), 215 with IF for CD8+ T cells (186 non-S/R and 29 S/R RCC), 15 kidney cancer cell lines with baseline RNA-sequencing (9 non-sarcomatoid and 6 sarcomatoid), and 20 kidney cancer cell lines with RNA-sequencing and drug sensitivity data for 437 therapeutic agents from CTRP v2. For the comparison of a dichotomous genomic feature (such as proportions of tumors that harbor mutations in a gene) between S/R and non-S/R, the observed sample size for DNA sequencing allows the detection of a difference of 10% or more in the rate of mutation between S/R and non-S/R with 80% power - assuming a mutation rate of 50% in non-S/R RCC, with a 2-sided type I error of 5% and a Fisher's exact test. While a larger sample size would have allowed the detection of smaller effect sizes, the incremental benefit of increasing the sample size becomes smaller at greater sample sizes. For instance, an additional 1000 patients (with the same current assumptions and power calculation parameters, including the same relative distribution between S/R and non-S/R RCC) would allow the detection of a difference in mutation rate between S/R and non-S/R RCC of 8.3% instead of 10%, representing a modest increase in the ability to detect smaller sample sizes.
For the in vitro cell line experiment, no sample size calculation was performed. The sample size used for in vitro experiment was consistent with those used in the field.

Data exclusions

All patients that had been consented for data collection, sequencing, and for which their sequencing had passed quality control were included.
For WES data from the CheckMate 010 and 025 cohorts, cross-sample contamination was assessed with the ContEst tool, and samples with ≥5% contamination were excluded. For RNA-sequencing data from the CheckMate 010 and 025 cohorts, samples were excluded if they had an interquartile range of log2(TPM+1)<0.5 or had less than 15,000 genes detected. Additionally, since the CheckMate cohort had been sequenced by a stranded protocol, samples were filtered if they had an End 2 Sense Rate<0.90 or End 1 Sense Rate>0.10 (as defined by RNA-seqQC2). For samples where RNA-seq was performed in duplicates, the run with a higher interquartile range of log2(TPM+1), considered a surrogate for better quality data, was used.

Replication

All in vitro cell viability assays were performed in 4 biological replicates.

Randomization

The Phase III trial (CheckMate 025) patients were equally randomized to nivolumab vs everolimus treatment arms. Randomization to this trial was stratified by MSKCC risk factors, geographic regions, and number of previous antiangiogenic therapies. The Phase II trial (CheckMate 010) patients were equally randomized 1:1:1 to 3 doses of nivolumab therapy and randomization was stratified by MSKCC risk factors and number of previous therapies.
The present analyses are secondary analyses of the data generated from these and other cohorts. Whenever applicable, confounding factors were taken into account in clinical outcomes comparisons (for overall survival, progression free survival, time to treatment failure, and overall response rate outcomes). The major confounding factors taken into account were IMDC or MSKCC risk factor groups, line of therapy, and background histology.
For the in vitro cell line experiment, cells from each cell line were randomly assigned to one of the four conditions (DMSO, alvocidib, SNS-032, and axitinib).

Blinding

Investigators were not blinded to patient allocation because all the clinical trials were open label trials.
Blinding is not applicable to the other retrospective cohorts, because these data are only observational and patient treatment was determined by treating physician.
For the cell line experiment, investigators were not blinded. Blinding was unnecessary because all data collection and analysis was performed uniformly across conditions.

Reporting for specific materials, systems and methods

We require information from authors about some types of materials, experimental systems and methods used in many studies. Here, indicate whether each material, system or method listed is relevant to your study. If you are not sure if a list item applies to your research, read the appropriate section before selecting a response.

Materials & experimental systems

n/a	Involved in the study
☒	☐ Antibodies
☐	☒ Eukaryotic cell lines
☒	☐ Palaeontology
☒	☐ Animals and other organisms
☐	☒ Human research participants
☐	☒ Clinical data

Methods

n/a	Involved in the study
☒	☐ ChIP-seq
☒	☐ Flow cytometry
☒	☐ MRI-based neuroimaging

Eukaryotic cell lines

Policy information about cell lines

Cell line source(s)	UOK127 and UOK276 were obtained from Dr. Linehan's laboratory at the National Cancer Institute (NCI) while RCJ41M, RCJ41T1, and RCJ41T2 were obtained from Dr. Ho's laboratory (Mayo Clinic, Phoenix, Arizona). Caki-1, Caki-2, A498, ACHN and 786-O were acquired from the American Type Culture Collection (ATCC). KMRC-1, KMRC-2, KMRC-20, VMRC-RCZ were obtained from JCRBbCell Bank and Sekisui XenoTech, LLC. BFTC-909 was obtained from Leibniz-Institut (DSMZ-Deutsche Sammlung von, Mikroorganismen und Zellkulturen GmbH).
Authentication	All commercially obtained cell lines were authenticated by karyotype analysis and DNA fingerprinting of short tandem repeats following manufactural instructions. Other cell lines were authenticated by immunohistology and genome mutation analysis.
Mycoplasma contamination	All cell lines were screened for mycoplasma contamination using the ATCC mycoplasma detection kit, which is a PCR-Based method. Negative Mycoplasma contamination was confirmed for all cell lines used in this study.
Commonly misidentified lines (See ICLAC register)	None

Human research participants

Policy information about studies involving human research participants

Population characteristics	All baseline characteristics for the patients included in this study are reported in the supplementary tables of the manuscript.
Recruitment	For the patients in the CheckMate 010 and 025 clinical trials, participants were recruited by local investigators at multiple cancer centers nationally and internationally. Patients were recruited from across the world. There is no evidence that these studies were prone to self-selection bias.<br>For the retrospective cohorts (DF/HCC and IMDC) patient data is collected sequentially,and therefore data collection is not prone to selection bias. For the DF/HCC cohort this data was collected from Dana-Farber Cancer Institute, Beth Israel Deaconess Medical Center, and Massachusetts General Hospital. For the IMDC cohort, data was collected from over 40 centers across the world.
Ethics oversight	All studies were approved by institutional review board or ethics committee at the cancer centers that enrolled patients and were conducted in accordance with Good Clinical Practice guidelines, as defined by the International Conference on Harmonisation.<br>Analysis was performed under a secondary use protocol, approved by the Dana-Farber Cancer Institute institutional review board.

Note that full information on the approval of the study protocol must also be provided in the manuscript.

Clinical data

Policy information about clinical studies

All manuscripts should comply with the ICMJE guidelines for publication of clinical research and a completed CONSORT checklist must be included with all submissions.

Clinical trial registration	CheckMate 010 (CM-010; NCT01354431) and CheckMate 025 (CM-025; NCT01668784)
Study protocol	Full trial protocol or relevant details are accessible online.
Data collection	Baseline, demographic, treatment, efficacy, follow-up, and other relevant data were collected at each center during each patients' visits and entered into a central database.<br><br>CheckMate 010 (CM-010; NCT01354431) enrolled all patients and collected follow-up data between May 2011 and January 2012 from 39 participating sites in the United States, Canada, Finland, and Italy.

CheckMate 025 (CM-025; NCT01668784) enrolled all patients and collected follow-up data from October 2012 through March 2014 from 146 sites in 24 countries in North America, Europe, Australia, South America, and Asia.

Outcomes	Choice of endpoints were in accordance with what is prevalent for this disease (advanced/metastastic RCC). The endpoints in each trial were assessed using the same disease evaluation criteria: Response Evaluation Criteria in Solid Tumors (RECIST version 1.1).
